# Calorie Restriction Impairs Anti-Tumor Immune Responses in an Immunogenic Preclinical Cancer Model

**DOI:** 10.3390/nu15163638

**Published:** 2023-08-18

**Authors:** Nguyen Tien Dung, Takeshi Susukida, Sisca Ucche, Ka He, So-ichiro Sasaki, Ryuji Hayashi, Yoshihiro Hayakawa

**Affiliations:** 1 Department of Medical Oncology, Toyama University Hospital, University of Toyama, Toyama 930-0194, Japan; bs.dungnt@gmail.com (N.T.D.); hsayaka@med.u-toyama.ac.jp (R.H.); 2 Section of Host Defences, Institute of Natural Medicine, University of Toyama, Toyama 930-0194, Japan; susukida@inm.u-toyama.ac.jp (T.S.); sisca.ucche@mail.ugm.ac.id (S.U.); d2168302@ems.u-toyama.ac.jp (K.H.); sasaki@inm.u-toyama.ac.jp (S.-i.S.)

**Keywords:** calorie restriction, host immune response, CD8^+^ T cell, immune checkpoint inhibitor

## Abstract

(1) Background: Although the important role of dietary energy intake in regulating both cancer progression and host immunity has been widely recognized, it remains unclear whether dietary calorie restriction (CR) has any impact on anti-tumor immune responses. (2) Methods: Using an immunogenic B16 melanoma cell expressing ovalbumin (B16-OVA), we examined the effect of the CR diet on B16-OVA tumor growth and host immune responses. To further test whether the CR diet affects the efficacy of cancer immunotherapy, we examined the effect of CR against anti-PD-1 monoclonal antibody (anti-PD-1 Ab) treatment. (3) Results: The CR diet significantly slowed down the tumor growth of B16-OVA without affecting both CD4^+^ and CD8^+^ T cell infiltration into the tumor. Although in vivo depletion of CD8^+^ T cells facilitated B16-OVA tumor growth in the control diet group, there was no significant change in the tumor growth in the CR diet group with or without CD8^+^ T cell-depletion. Anti-PD-1 Ab treatment lost its efficacy to suppress tumor growth along with the activation and metabolic shift of CD8^+^ T cells under CR condition. (4) Conclusions: Our present results suggest that a physical condition restricted in energy intake in cancer patients may impair CD8^+^ T cell immune surveillance and the efficacy of immunotherapy.

## 1. Introduction

The role of physical condition, including energy/food intake, diet, exercise, and weight, has been studied extensively in cancer progression. Diets high in calories, saturated fats, and refined sugars are associated with increased cancer risk and faster tumor growth, while diets high in fruits, vegetables, and whole grains are associated with reducing the risk of cancer and improving cancer outcomes [1,2,3,4]. Regular exercise has been shown to reduce inflammation, improve immune function and hormone regulation, and slow cancer growth [5,6]. In contrast, obesity is known to be associated with an increased risk of certain types of cancer and support aggressive tumor growth [7,8]. 

The correlation between physical condition and host immunity has also been studied recently. It has been reported that malnutrition and an unhealthy diet, characterized by a low intake of fruits, vegetables, and micronutrients, impair immune function and increase susceptibility to infections [9,10]. On the other hand, a diet rich in nutrients and phytochemicals, such as flavonoids, carotenes, and vitamins, is known to increase immune function and reduce inflammation [11,12]. Physical activity has also been shown to improve immune function by enhancing immune cell circulation and reducing inflammation [6,13]. Contrarily, obesity and a sedentary lifestyle were reported to impair immune function and increase the risk of infection [14,15]. Recent findings suggest the effect of dietary calorie intake has significant impact on cancer disease and therapy [16,17,18,19]. In general, calorie restriction (CR) is defined as the 20% to 40% reduction of the average daily caloric intake without incurring malnutrition or the deprivation of essential nutrients, and has been reported to affect cancer prevention and therapy [16,17,18,19].

In this study, we aim to investigate the effect of calorie restriction (CR) on anti-tumor immune responses in an immunogenic preclinical cancer model. We conducted a preclinical study using an immunogenic tumor model with B16 melanoma expressing ovalbumin (B16-OVA) and tested for the effect of CR on the host anti-tumor immune responses. B16-OVA model is one of the most well-established models used to monitor host CD8^+^ T cell-dependent immune response. In addition, the effect of CR on immunotherapy with immune checkpoint blockade (ICB) was studied. Although the CR diet delayed tumor growth, the host anti-tumor immunity and the response to anti-PD-1 treatment were poor due to the alteration of CD8^+^ T cells’ number and function. These findings may contribute to understanding the response to ICB under energy restriction in cancer patients.

## 2. Materials and Methods

### 2.1. Cells

Murine melanoma cell line constitutively expressing ovalbumin, B16-OVA (MO4), was a kind gift from Dr. Shinichiro Fujii (Riken, Japan). Cells were cultured using Eagle’s Minimum Essential Media (EMEM; Nissui, Tokyo, Japan) and supplemented with 2 mM l-glutamine, 10% fetal bovine serum, 100 U/mL penicillin, and 100 µg/mL streptomycin. Cells were all maintained at 37 °C in a humidified atmosphere of 95% air/5% CO_2_.

### 2.2. Animals and Diet

All experiments were conducted using 7-week-old C57BL/6 mice which were purchased from CLEA Japan, Inc. (Tokyo, Japan). Mice were randomized into 2 groups using ad lib diet/CR diet, then each group was divided into 2 cages, with/without vehicles, and were group-housed and acclimatized to the animal facility environment for 7 days before experimentation, after which we measured daily food intake on average over the next 3 days. Animal rooms were maintained at 22.2  ±  1 °C and 30–70% humidity with a 12 h day/light cycles. On day 0, mice were subcutaneously inoculated with B16-OVA melanoma cells (10^5^), then the CR group started fasting. Purified Rodent Diet with 10% fat (diet #D12450J, Research Diets, Inc., New Brunswick, NJ, USA) were fed for the entirety of the study, except a diet with 40% CR was customized and used for the CR group (diet # D16042001, Research Diets, Inc.). Tumors and body weight were measured 3 times weekly. Tumor growth was measured by a caliper square measuring along the longer axes (a) and the shorter axes (b) of the tumors. Tumor volumes (mm^3^) were calculated by the formula: tumor volume (mm^3^) = ab2/2. Tissues were collected between 8 a.m. and 12 p.m., ~14 h after the last meal. All experiments were approved (Animal Experiment Protocol: A209INM-4, A2022INM-5) and performed in accordance with the guidelines of Care and Use of Laboratory Animals of University of Toyama.

### 2.3. Antibody Treatment

For CD8^+^ T cell depletion, B16-OVA-inoculated mice were pretreated with anti-CD8 antibody (clone 53.6.2, Bio X Cell, Lebanon, NH, USA) (0.25 mg/mouse i.p.) 2 times at 2 days prior day 0, and then again at days 7 and 14. For PD-1 blocking, B16-OVA-inoculated mice were treated with anti-PD-1 antibody (RMP1–14, BioXCell) (0.2 mg/mouse i.p.) at days 3, 6, and 9 from day 0.

### 2.4. Tumor-Infiltrating Lymphocyte (TIL) Isolation and Flow Cytometry

Tumor samples were cut into small pieces and digested in serum-free RPMI 1640 medium containing 2 mg/mL collagenase (Roche Diagnostics GmbH) and 0.1 mg/mL DNase I (Roche Diagnostics GmbH) for 1 h at 37 °C. The cells were then incubated with a saturating amount of fluorophore-conjugated antibodies against PE-Cy/7 CD45 (30-F11), FITC CD3ε (145-2C11), PerCP-Cy5.5 CD4 (GK1.5), APC CD8 (53-6.7), PE CD44 (IM7), APC-Cy7 CD62L (MEL-14) were purchased from Biolegend (San Diego, CA, USA). FACS Canto II (BD Biosciences, Franklin Lakes, NJ, USA) was used for FACS analysis and data were analyzed using FlowJo software v.10 (BD Biosciences).

### 2.5. Real-Time CD8^+^ T Cell Metabolic Analysis 

After euthanizing the mice by cervical dislocation, cells were isolated by tumor-draining lymph node (tLN) from the mice fed with either a normal diet or CR diet. CD8^+^ T cells were negatively selected using MojoSort^TM^ mouse CD8 T cell Isolation Kit (BioLegend) according to the manufacturer’s protocol. The isolated CD8+ T cell were seeded at the density of 5 × 10^5^ cells/well in Poly-D-Lysine (Thermo Fisher Scientific, Waltham, MA, USA)-coated XFe24 plates in 500 μL Seahorse XF RPMI medium containing 1 mM pyruvate, 2 mM glutamine, and 10 mM glucose. ECAR and OCR were measured on an XFe24 Extracellular Flux analyzer (Agilent Technologies, Santa Clara, CA, USA) using the Seahorse XF Cell Mito Stress test kit (Agilent Technologies). The OCR and ECAR values were obtained at baseline and after the injections of oligomycin (final concentration 1 μM), FCCP (final concentration 1 μM), and antimycin/rotenone (final concentration 0.5 μM), respectively.

### 2.6. Statistical Analysis

Statistical analyses were performed using GraphPad Prism 8 Software (GraphPad Software, La Jolla, CA, USA). Significance was determined using either Bonferroni’s test for multiple comparisons following one-way ANOVA or the unpaired two tailed t-test (Student’s *t*-test). In all cases, *p* values of <0.05 were considered statistically significant. All data were obtained from the groups of 4–10 mice. 

## 3. Results

### 3.1. Effect of CR on Host Anti-Tumor Immunity

**Figure 1:**
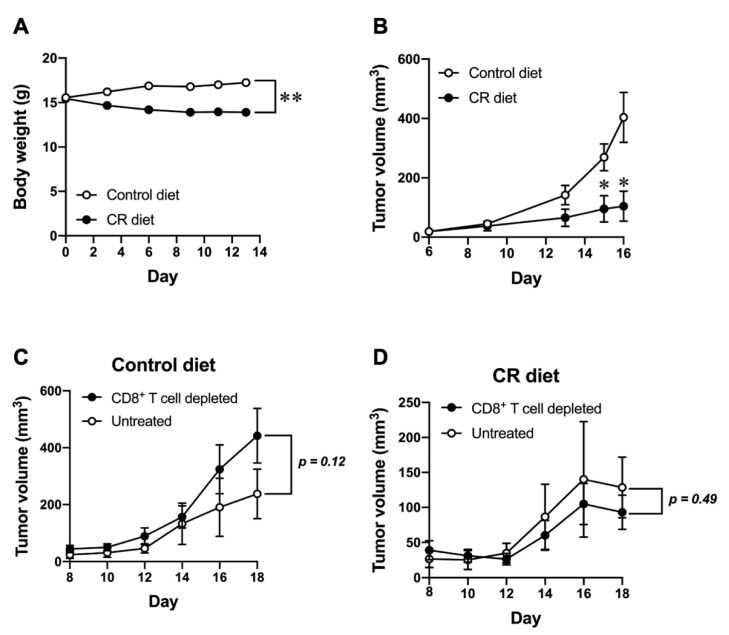
Effect of CR on tumor growth in the presence or absence of host CD8^+^ T cell. (**A**) Body weight change in in control-diet-fed and CR-diet-fed C57BL/6J mice. (**B**) B16-OVA tumor size progression in control-diet-fed and CR-diet-fed C57BL/6J mice. (**C**,**D**) B16-OVA tumor size progression in control-diet-fed (**C**) and CR-diet-fed (**D**) mice with/without CD8^+^ T cell depletion. Each plot represents the mean ± SEM (*n* = 4–21); * *p* < 0.05, ** *p* < 0.01, compared with control-diet-fed mice or antibody untreated group; indicated *p* values were obtained from a statistical comparison, the unpaired two tailed *t*-test (Student’s *t*-test).

We first examined the effect of CR on B16-OVA tumor growth. Mice were subcutaneously inoculated with B16-OVA cells, then subjected to a 40% CR condition from the day of tumor inoculation and monitored for tumor growth. The body weight change in CR-diet-fed mice is shown in Figure 1A by comparing control-diet-fed mice. There was a substantial decrease in the body weight in CR diet group, however, it was no more than 20% reduction and was reversible by regular feeding. As shown in Figure 1B, the growth of tumors was significantly impaired in mice fed with the CR diet compared to controls. To further determine the contribution of the host immune response, particularly CD8^+^ T cells, for controlling tumor growth in mice fed with the control diet or the CR diet, mice were treated with anti-CD8 Ab to deplete CD8^+^ T cells. In the control-diet-fed mice, anti-CD8 Ab treatment showed a trend to increase the growth of B16-OVA tumor (Figure 1C); however, it did not show any different tumor growth in the CR-diet-fed mice (Figure 1D). These results potentially implicate that the CR diet retards the growth of B16-OVA tumor. Although there were no statistically significant differences in both CD8+ T cell-depleted control diet and CR diet groups, the CR diet might also has a negative impact on the host anti-tumor immunity by CD8^+^ T cells in controlling tumor growth.

### 3.2. Effect of CR on the Responsiveness to Immune Checkpoint Blockade

**Figure 2:**
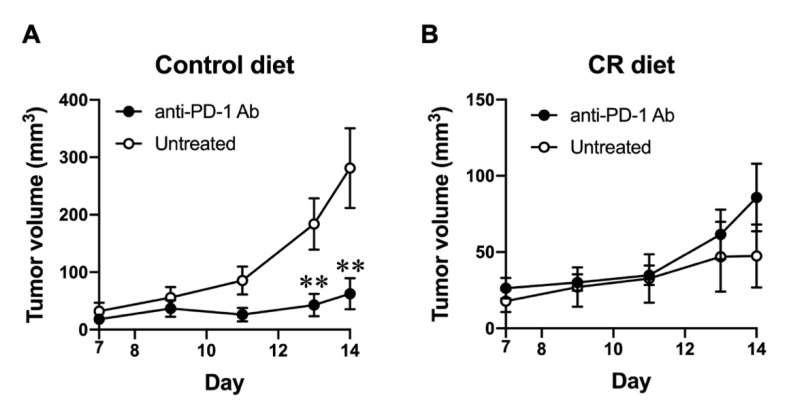
Effect of CR on the responsiveness to immune checkpoint blockade. (**A**,**B**) B16-OVA tumor size progression in control-diet-fed (**A**) and CR-diet-fed (**B**) C57BL/6J mice with/without anti-PD-1 antibody treatment (0.2 mg/mouse i.p.) at days 3, 6, and 9 from day 0. Each plot represents the mean ± SEM (*n* = 4–21); ** *p* < 0.01, compared with control-diet-fed mice or antibody-untreated group; indicated *p* values were obtained from a statistical comparison, the unpaired two tailed *t*-test (Student’s *t*-test).

As CR-impaired host CD8^+^ T cells display immunity against the B16-OVA tumor, we next tested whether CR affects the responsiveness to immune checkpoint blockade in tumor-bearing mice. B16-OVA tumor-bearing mice, either fed with the control diet or a 40% CR diet, were treated with anti-PD-1 Ab after the tumor inoculation. As shown in Figure 2A, anti-PD-1 Ab treatment significantly inhibited the growth of B16-OVA tumor in control-diet-fed mice. Contrarily, anti-PD-1 Ab treatment did not show any effect on the growth of the B16-OVA tumor in CR-fed-mice (Figure 2B), suggesting CR has a negative effect on the responsiveness to immune checkpoint blockade, presumably through a CD8^+^ T cell-dependent anti-tumor immune response. 

### 3.3. Effect of CR on the Population of Tumor-Infiltrating Lymphocytes (TILs)

**Figure 3:**
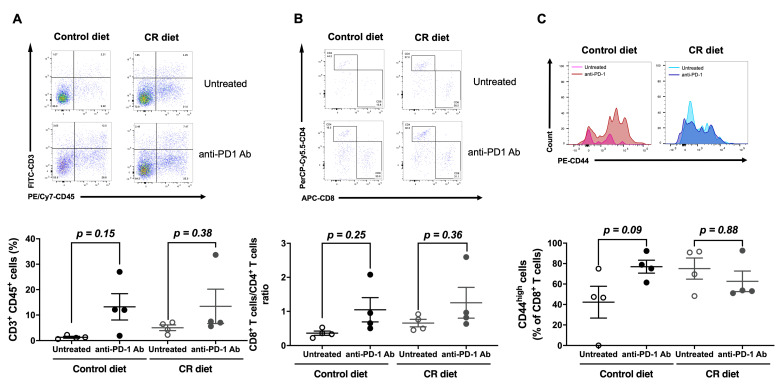
Effect of CR on the population of tumor-infiltrating lymphocytes (TILs). (**A**) Representative dot plots depicting (upper panels) or percentages (lower panel) of TILs expressing CD3 and CD45. B16-OVA-inoculated mice were treated with/without anti-PD-1 antibody (0.2 mg/mouse i.p.) at days 3, 6, and 9 from day 0. (**B**) Representative dot plots depicting (upper panels) or percentages (lower panel) of cells expressing CD4 and CD8 in the gated CD3^+^CD45^+^ TILs. (**C**) Representative histograms representing surface expression (upper panels) or median fluorescence intensity (MFI) values (lower panel) of CD44 on CD3^+^CD8^+^CD45^+^ TILs derived from B16-OVA-inoculated mice. Each plot represents the mean ± SEM (*n* = 4); indicated *p* values were obtained from a statistical comparison; one-way ANOVA with Bonferroni’s multiple comparisons correction.

In order to understand the effect of CR on CD8^+^ T cell-dependent anti-tumor immune responses, we next examined the population of TILs in B16-OVA tumor-bearing mice fed with the control diet or the CR diet, and with or without anti-PD-1 Ab treatment. B16-OVA inoculated mice were fed with the control diet or the CR diet from day 0, and then treated with or without anti-PD-1 Ab (on days 3, 6, and 9). The tumor samples were collected on day 16 to isolate TILs, and subjected to flow cytometry analysis. There was no significant difference in the tumor infiltration of CD3^+^ T cells (Figure 3A), the ratio of CD8^+^/CD4^+^ T cells (Figure 3B), or CD44high effector CD8^+^ T cells (Figure 3C) between control-diet- and CR-diet-fed mice. In control-diet-fed mice, anti-PD-1 Ab treatment increased the population of those CD8^+^ T cells in line with inhibiting the B16-OVA tumor growth (Figure 3). Although anti-PD-1 Ab treatment did not show an anti-tumor effect, it had a similar effect on the population of tumor-infiltrating CD8^+^ T cells in CR-diet-fed mice (Figure 3). These results suggest that CR may not impair the anti-tumor effect of anti-PD-1 Ab treatment by affecting the presence of tumor-infiltrating effector CD8^+^ T cells. 

### 3.4. Effect of CR on the Metabolic Status of CD8^+^ T Cell in B16-OVA Tumor-Bearing Mice

**Figure 4:**
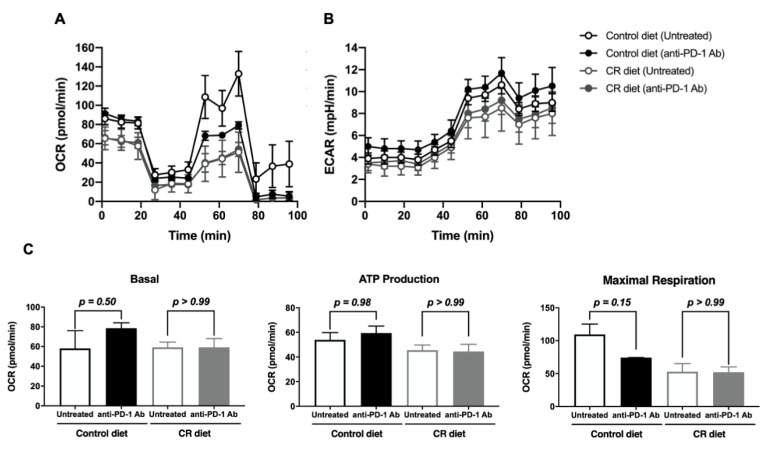
Effect of CR on the metabolic status of CD8^+^ T cell in B16-OVA tumor-bearing mice. (**A**,**B**) The oxygen consumption rate (OCR) (**A**), the extracellular acidification rate (ECAR) (**B**), and the summary of respiration status and ATP production (**C**) of the isolated CD8^+^ T cells from lymph nodes of each group was measured using a Seahorse XFe24 analyzer. Each plot represents the mean ± SEM (*n* = 3–4).

Considering that there was no significant effect of CR diet on the population of TILs, we sought to understand the metabolic status of CD8^+^ T cells in B16-OVA tumor-bearing mice fed with the control or the CR diet, and also compared their response to anti-PD-1 Ab treatment using an extracellular flux analyzer. In CR-diet-fed mice, CD8^+^ T cells in the B16-OVA tumor-draining lymph node (tLN) showed lower oxygen consumption rates (OCR, Figure 4A) and extracellular acidification rates (ECAR, Figure 4B) compared to control diet fed mice, suggesting the CR diet generally reduces the metabolic activity of CD8^+^ T cells compared to the control diet. Upon anti-PD-1 Ab treatment, CD8^+^ T cells in tLN of control-diet-fed mice showed lower OCR and higher ECAR compared to the untreated group, whereas those of CR-diet-fed mice did not show any difference (Figure 4). These results indicate that anti-PD-1 treatment activated and induced a metabolic shift of CD8^+^ T cells to the glycolytic pathway under control-fed conditions; however, it cannot be recapitulated in CR-diet-fed mice. 

## 4. Discussion

To understand the importance of physical condition, particularly energy intake, in cancer progression, we studied the effect of CR on anti-tumor immune responses in the immunogenic B16-OVA melanoma model. CR significantly slowed down the tumor growth of B16-OVA without affecting both CD4^+^ and CD8^+^ T cell infiltration into the tumor. While the in vivo depletion of CD8^+^ cells accelerated B16-OVA tumor growth in the normal diet group, there was no significant change in the tumor growth of the CR group with or without CD8^+^ cells. Considering anti-PD-1 Ab lost its efficacy to suppress tumor growth under the CR condition along with the alteration of CD8^+^ T cells’ mitochondrial activity, the energy restricted physical condition in cancer patients may impair CD8^+^ T cell immune surveillance and the efficacy of immunotherapy.

It has been known that CR reduces cancer risk and improves outcomes in preclinical and clinical studies. Calorie restriction activates molecular pathways that enhance cellular defenses, promote DNA repair, and reduce oxidative damage, which may contribute to its anticancer effects [20,21]. Additionally, CR has been shown to enhance the effectiveness of cancer treatments such as chemotherapy and radiation [22,23]. Indeed, some studies have demonstrated that calorie restriction can improve immune function and reduce inflammation, potentially contributing to increased health [21,24,25]. Several studies have also provided evidence indicating that calorie restriction can enhance immune function and alleviate inflammation, leading to potentially improved overall health [26,27]. CR has been shown to prevent mitochondrial dysfunction and enhance mitochondrial efficiency by reducing oxidative stress and inflammation, promoting mitochondrial biogenesis, and improving mitochondrial quality control mechanisms. Therefore, CR may improve cellular metabolism and energy production, reduce cellular damage, and contribute to improved health and longevity [28,29]. 

Contrary to the role of CR in cancer suppression, patients with sarcopenia or cachexia resulting from chronic caloric deficits may have a poorer response to immunotherapy, lower progression-free survival, and lower overall survival rates, according to some studies [30,31]. In line with our findings, those patients also showed a reduction in immune cell infiltration into the tumor microenvironment and impaired T cell activation [30,31]. In general, CD8^+^T cells use glycolysis during their differentiation to effector cells, and PD-1 ligation increases fatty acid oxidation (FAO) [32]. The mitochondrial activation of CD8+ T cells has been reported to enhance the efficacy of PD-1 blockade [33,34]. Therefore, it must be critical to balance between calorie restriction and maintaining an adequate calorie intake to avoid negative impacts on immune function and the subsequent response to cancer therapy.

## 5. Conclusions

In this study, we aim to investigate the effect of calorie restriction (CR) on anti-tumor immune responses in an immunogenic preclinical cancer model. Although the CR diet delayed tumor growth, the effect of anti-PD-1 treatment was poorer in CR mice. Although our presented study is still exploratory with a relatively low sample size and large data variations, our present results suggest that the energy restricted physical condition of cancer patients may impair CD8^+^ T cell immune surveillance and the efficacy of immunotherapy.

## Data Availability

The data presented in this study are available on request from the corresponding author.
